# ROP18-Mediated Transcriptional Reprogramming of HEK293T Cell Reveals New Roles of ROP18 in the Interplay Between *Toxoplasma gondii* and the Host Cell

**DOI:** 10.3389/fcimb.2020.586946

**Published:** 2020-11-30

**Authors:** Jie-Xi Li, Jun-Jun He, Hany M. Elsheikha, Jun Ma, Xiao-Pei Xu, Xing-Quan Zhu

**Affiliations:** ^1^ State Key Laboratory of Veterinary Etiological Biology, Key Laboratory of Veterinary Parasitology of Gansu Province, Lanzhou Veterinary Research Institute, Chinese Academy of Agricultural Sciences, Lanzhou, China; ^2^ Faculty of Medicine and Health Sciences, School of Veterinary Medicine and Science, University of Nottingham, Loughborough, United Kingdom; ^3^ Heilongjiang Key Laboratory for Zoonosis, College of Veterinary Medicine, Northeast Agricultural University, Harbin, China; ^4^ College of Veterinary Medicine, Shanxi Agricultural University, Taigu, China

**Keywords:** ROP18, transcriptome, *Toxoplasma gondii*, differentially expressed genes, transcription factors

## Abstract

*Toxoplasma gondii* secretes a number of virulence-related effector proteins, such as the rhoptry protein 18 (ROP18). To further broaden our understanding of the molecular functions of ROP18, we examined the transcriptional response of human embryonic kidney cells (HEK293T) to ROP18 of type I *T. gondii* RH strain. Using RNA-sequencing, we compared the transcriptome of ROP18-expressing HEK293T cells to control HEK293T cells. Our analysis revealed that ROP18 altered the expression of 750 genes (467 upregulated genes and 283 downregulated genes) in HEK293T cells. Gene ontology (GO) and pathway enrichment analyses showed that differentially expressed genes (DEGs) were significantly enriched in extracellular matrix– and immune–related GO terms and pathways. KEGG pathway enrichment analysis revealed that DEGs were involved in several disease-related pathways, such as nervous system diseases and eye disease. ROP18 significantly increased the alternative splicing pattern “retained intron” and altered the expression of 144 transcription factors (TFs). These results provide new insight into how ROP18 may influence biological processes in the host cells *via* altering the expression of genes, TFs, and pathways. More *in vitro* and *in vivo* studies are required to substantiate these findings.

## Introduction

*Toxoplasma gondii* is an opportunistic and obligate intracellular protozoan, which can establish a persistent infection (Sibley, 2003). *T. gondii* infects nearly one third of the world’s human population (Tenter et al., 2000). Strains of *T. gondii* are categorized into three major genotypes based on their virulence in mice into types I, II, and III. Genotype I strains are highly virulent, whereas strains of genotypes II and III are less virulent (Saeij et al., 2006). In general, infection of immunocompetent individuals is either asymptomatic or causes mild flu-like symptoms (Beazley and Egerman, 1998; Schneider et al., 2013). High risks of encephalitis and even death due to reactivation of a latent infection can occur in immuno-compromised individuals (Dubey, 2004; Weiss and Dubey, 2009; Kaye, 2011; An et al., 2018). *T. gondii* can also result in adverse health consequences in congenitally infected fetuses (Elsheikha, 2008).

In order to establish an infection, *T. gondii* manipulates the host cells *via* altering the cellular metabolism (Ma et al., 2019), dysregulating the gene expression (He et al., 2016), and subverting the immune response (Yarovinsky, 2014). Infection of *T. gondii* elicits the production of interferon gamma (IFN-γ), tumor necrosis factor (TNF), interleukin 10 (IL-10), IL-12, and several cytokine receptors (Gazzinelli et al., 1996; He et al., 2016), while reduces production of nitric oxide (Rozenfeld et al., 2005). The parasite performs these functions by secreting a number of effector molecules into host cell, such as dense granule proteins (GRAs) and rhoptry proteins (ROPs) (Bradley and Sibley, 2007) that play important roles in the regulation of immune responses (Fox et al., 2016) and gene expression (Rastogi et al., 2020). For example, GRA15 regulates the expression of genes in the NF-κB pathway (Sangare et al., 2019); ROP17 inhibits the expression of innate immune response genes (Li et al., 2019). ROP18 induces apoptosis in mouse neuroblastoma Neuro2a cells *via* endoplasmic reticulum stress-mediated apoptosis pathway (Wan et al., 2015) and inhibits the differentiation of cultured murine neural stem cells *via* inhibiting the activity of the Wnt/β-catenin signaling pathway (Zhang et al., 2017).

ROP18 is serine/threonine phosphokinase and contributes to the virulence of *T. gondii* (Hunter and Sibley, 2012). The expression of ROP18 is higher in *T. gondii* genotype I strain than in genotype III strain (Taylor et al., 2006). Deletion of ROP18 significantly increases the survival of infected mouse (Behnke et al., 2015). *T. gondii* utilizes ROP18 to prevent disruption of parasitophorous vacuole membrane (PVM) *via* phosphorylating the immunity-related GTPases (IRGs) of host cell, and to regulate the biological processes of neurocytes (Steinfeldt et al., 2010; Fleckenstein et al., 2012; Wan et al., 2015; Zhang et al., 2017). Also, ROP18 *via* degradation of the transcription factor (TF) p65 inhibits the NF-κB pathway and suppresses the inflammatory responses to promote its own survival and growth (Du et al., 2014). Besides p65, ROP18 also targets other TFs, such as p53 and Smad1 (Yang et al., 2017).

These diverse functions of ROP18 have led us to hypothesize that ROP18 exerts its multiple effects *via* reprogramming host cell transcriptome. In the present study, we investigated the molecular involvement and significance of ROP18 in the pathogenesis of *T. gondii* infection by investigating the influence of ROP18 on the transcriptome of HEK293T cells using RNA sequencing (RNA-Seq).

## Materials and Methods

### Cell Culture and Parasite

HEK293T (human embryonic kidney) cells were purchased from the American Type Culture Collection (ATCC, Manassas, VA) and were cultured in high glucose Dulbecco’s modified Eagle’s medium (Sigma-Aldrich, USA), containing 2 mM l-glutamine, 100 U/ml penicillin and 10 mg/mL streptomycin, and 10% (vol/vol) fetal bovine serum (Gibco, New Zealand). The cultured HEK293T cells were incubated at 37°C in humidified air with 5% CO_2_. HEK293T cell line was chosen in this study due to its high efficiency for transfection and expression of exogenous genes. *T. gondii* RH strain was maintained *via* passage in human foreskin fibroblast (HFF) cells. Total RNA of the *T. gondii* RH tachyzoites was extracted using TRIzol reagent (Invitrogen, USA) according to the manufacturer’s protocol. The residual genomic DNA of *T. gondii* was removed using RNase-Free DNase (Ambion, Shanghai, China).

### Plasmid Construction

The coding sequence (CDS) of ROP18 of *T. gondii* RH strain (GenBank No. JX045330) was amplified from total RNA extracted from tachyzoite of *T. gondii* RH strain using the primers: ROP18-F (5’-GGGGGATCCATGACACTTGGTCCTTCAAAACTCG-3’) and ROP18-R (5’-GGGGTCGACTTCTGTGTGGAGATGTTCCTGCTGTTC-3’). The PCR conditions were set as follows: pre-denaturation for 5 min at 98°C followed by 35 cycles of 98°C for 20 s, 56°C for 18 s, and 72°C for 30 s; 72°C for 5 min and hold at 4°C. The PCR product was purified using Gel Extraction kit (OMEGA, China). The purified ROP18 CDS was cloned into PCMV-N-HA vector using BamHI and SalI restriction enzymes (NEB, USA), according to the manufacturer’s instructions. The constructed plasmid (PCMV-N-HA-ROP18) was transformed into *E. coli* DH5α competent cells (TIANGEN, China). Single bacterial colony was randomly selected and identified using PCR primers ROP18-F and ROP18-R. Positive colonies were sequenced by Genscript Corporation (Nanjing, China). The plasmid of PCMV-N-HA-ROP18 bacterial colony was extracted using Endofree Plasmid Kit (TIANGEN, China) following the manufacturer’s instructions, and the extracted plasmid was stored at −20°C until use.

### Transfection of HEK293T Cells

The HEK293T cells were cultured in T-25cm^2^ cell culture flasks (NEST, China). When the monolayers reached 70%–80% confluence, transfection was performed using Xfect™ Transfection Reagent (Takara, China). Briefly, 30 µg PCMV-N-HA-ROP18 and PCMV-N-HA (empty control vector) were diluted separately in 300 µl Xfect™ transfection buffer. Then, 10 µl Xfect™ polymer was added and vortexed for 5 s at high speed, followed by incubation for 10 min at room temperature. The mixture was then added into the supernatant of the cultured cells and incubated for 4 h. Following the incubation, the DMEM of transfected cell was replaced with 5 ml fresh DMEM supplemented with 10% FBS. Forty-eight hours post transfection, transfected cells were collected and used for Western blotting, indirect immunofluorescence and transcriptome analysis as described below.

### Western Blotting

We examined whether ROP18 was correctly expressed in HEK293T cells using Western blotting analysis. Briefly, total protein was extracted using ProteinExt™ Mammalian Total Protein Extraction Kit (TRAN, China). Then, 20 µg of the extracted protein and 10 µl PageRuler™ Prestained Protein Ladder (Thermo Scientific, USA) were electrophoresed on 12% ExpressplusTM PAGE Gels (GenScript, China) under 120V and then electrotransferred to PVDF membrane (Thermo, Germany). The PVDF blotting membrane was incubated with anti-HA tag antibody (Abcam, UK) overnight at 4°C. Then, the PVDF membrane was washed three times with 1× TBS (Solarbio, China) and the PVDF membrane was incubated with secondary antibody, goat anti-mouse IgG H&L (HRP) (Abcam, UK), for 1 h at 37°C. The PVDF membrane was washed three times by 1× TBS. The ECL reagent (Solarbio, China) was used to detect the targeted protein (Solarbio, China). The Western blot image was recorded by Gel DocTM XR+ with image lab™ Software (BIO-RAD, USA).

### Indirect Immunofluorescence Assay

The transfected cells were washed three times with phosphate buffered saline (PBS) and fixed with 4% paraformaldehyde (Solarbio, China) for 10 min. The paraformaldehyde was discarded and the fixed cells were washed three times with PBS, permeabilized using 0.1% Triton X-100 (Beyotime, China), and blocked with 5% bovine serum albumin for 1 h. Following three times washing with PBS, primary mouse anti-HA tag antibody (Abcam, UK) was used to recognize HA tag of ROP18 protein. After incubation with the anti-HA tag antibody at 4°C overnight, the residue HA-tag antibody was discarded and the fixed cells were washed three times with PBS, and then incubated with goat anti-mouse IgG H&L conjugated with Alexa Fluor^®^555 (Abcam, UK) at 37°C for 1 h. Nucleus was counter-stained with 10 µg/ml DAPI (Solarbio, China). Before the immunofluorescence detection, the goat anti-mouse IgG H&L antibody and DAPI were discarded by washing three times with PBS. The immunofluorescence images were recorded using a Fluorescence microscope Axiovert 100TV (Zeiss, Germany).

### Total RNA Extraction and RNA Sequencing of HEK293T Cells

Total RNA of HEK293T cells was extracted by using TRIzol Reagent (Invitrogen China Ltd, Beijing, China) according to the manufacturer’s instructions. All extracted RNA samples were treated with RNase-Free DNase (Ambion, Shanghai, China) to remove residual genomic DNA. The concentration and quality of RNA were detected using the Agilent 2100 Bioanalyzer (Agilent Technologies, Palo Alto, Calif.). mRNA was isolated from total RNA using Poly-T oligo-conjugated magnetic beads, and then mRNA was reversely transcribed into cDNA with PrimerScriptTMRT reagent kit with gDNA Eraser (Takara, China) following the manufacturer’s instructions. Construction of transcriptomic libraries and RNA-Seq were performed by BGI-Shenzhen (Shenzhen, China).

### Sequencing Quality and Mapping of Sequencing Reads

Reads were trimmed to remove the adaptor primers, low-quality reads, and very short (<50 nt) reads. The quality of RNA-Seq was checked by using the quality scores Q20 and Q30. The clean reads were mapped against the human reference genome (ftp://ftp.ncbi.nlm.nih.gov/genomes/H_sapiens/current/GCF_000001405.39_GRCh38.p13/) using SOAPaligner/SOAP2 software. Reads per kilobase per million mapped reads (RPKM) method was used for calculation of the relative gene expression (Mortazavi et al., 2008). rMATS software was used to detect gene alternative splicing (AS) events among samples, including skipped exon (SE), alternative 5’ splicing site (A5SS), alternative 3’ splicing site (A3SS), mutually exclusive exons (MXE) and retained intron (RI).

### Bioinformatic Analysis of the Differentially Expressed Genes

DESeq2 software was used to determine gene expression and identify differentially expressed genes (DEGs) between the PCMV-N-HA-ROP18 transfected cells and PCMV-N-HA transfected (control) cells. The Benjamini and Hochberg false discovery rate (FDR) was used to correct multiple hypothesis testing *P* values. Genes with FDR adjusted *P* values of Fisher’s exact test ≤ 0.05 and | log_2_(fold change) | ≥ 1 were deemed as DEGs. The fold change (FC) = gene RPKM value of ROP18-expressing HEK293T cells/gene RPKM value of control-HEK293T cells. The gene expression data were clustered using Euclidean distance. The functional annotation and pathways involving the DEGs were analyzed using Gene Ontology (GO), Reactome, and KEGG (http://www.kegg.jp/) pathway enrichment analyses. Fisher’s exact test adjusted with FDR was used to identify significantly enriched GO terms or pathways. The FDR adjusted *P* value ≤ 0.05 was used to identify the significantly enriched GO terms or pathways. TRRUST database was used to identify the relationship between TFs and their target genes. Cytoscape software was used to visualize the relationship between DEGs, GO terms, and pathways.

### Real-Time Quantitative PCR (qRT-PCR) Validation

We examined the reliability of RNA-seq results by using qRT-PCR. Twenty DEGs were chosen, including *WNK4*, *TNC*, *TNFRSF9*, *IL6R*, *PCK1*, *FRMD1*, *TES1*, *INHBA*, *CD44*, *LINC01599*, *LOC400710*, *EIF4EBP3*, *LOC101929181*, *OR2B6*, *LRRC46*, *FGF21*, *KRTAP5*-*2*, *KCNN4*, *SEZ6*, and *RNU1*-*2*. *GAPDH* was included as a reference gene. The details of all the primers are shown in **Supplementary Table S1**. Briefly, total RNA was extracted from the transfected cells, and reverse transcribed into cDNA using PrimerScriptTMRT reagent kit with gDNA Eraser (Takara, China). The cDNA was stored at –80°C until use. The following qRT-PCR conditions were used for gene amplification: 95°C for 10 min, followed by 40 cycles of denaturing at 94°C for 15 s and 60°C for 1 min. The melt curve analysis ranged from 72°C to 95°C to ensure that specific product was amplified in each qRT-PCR reaction. The 2^−ΔΔCT^ relative expression calculation method was used to calculate the relative gene expression levels of the examined genes (Livak and Schmittgen, 2001).

## Results

### Confirmation of ROP18 Expression in HEK293T Cell

**Figure 1:**
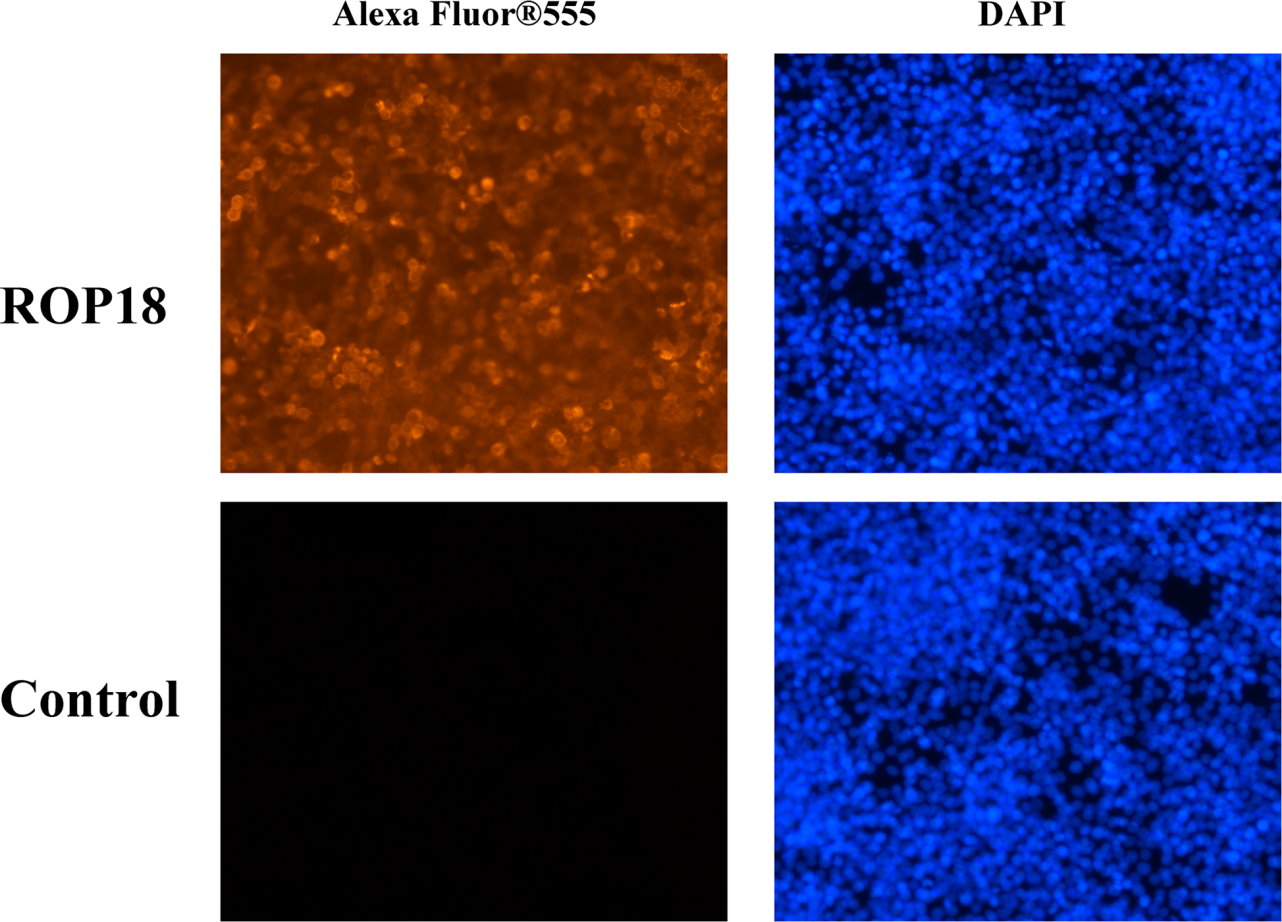
Indirect immunofluorescence of the transfected HEK293T cells. The ROP18 protein tagged with HA was stained with AlexaFluor 555 (Orange) and the nucleus was counterstained with DAPI (Blue). The HEK293T cells transfected with PCMV-N-HA-ROP18 showed high density of orange signal, whereas HEK293T cells transfected with PCMV-N-HA did not show any fluorescent signal.

Sequencing of PCMV-N-HA-ROP18 showed that the CDS of ROP18 of *T. gondii* RH strain had been correctly cloned into the PCMV-N-HA plasmid. The results of Western blotting demonstrated that ROP18 protein was correctly expressed in HEK293T cells; however, no protein was detected in the HEK293T cells transfected with PCMV-N-HA (**Figure S1**). The efficiency of transfection was examined using indirect immunofluorescence analysis, which demonstrated the high expression of ROP18 in HEK293T cells. As expected, no fluorescent signal was detected in HEK293T cells transfected with PCMV-N-HA (**Figure 1**).

### RNA-Sequencing and Identification of Differentially Expressed Genes

**Figure 2:**
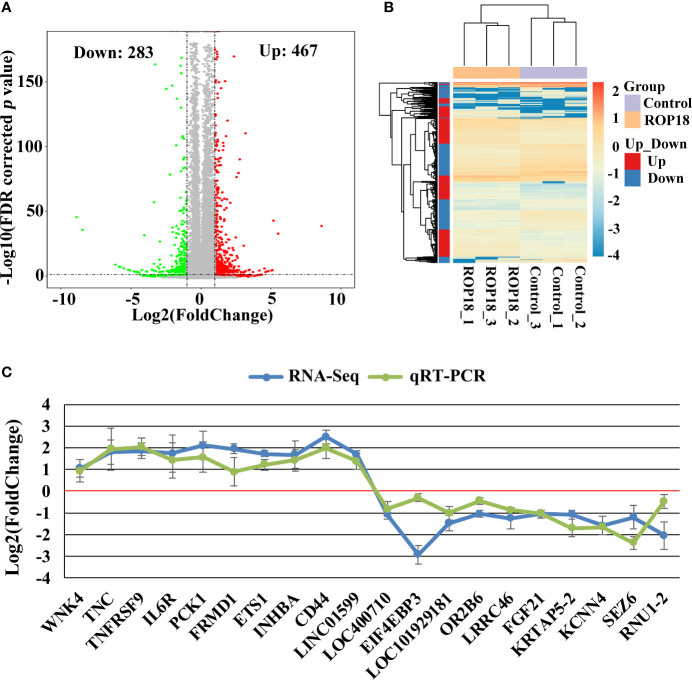
Differentially expressed genes (DEGs) and qRT-PCR validation. **(A)** Volcano plot showing gene expression changes in ROP18-expressing HEK293T cells, including 467 upregulated genes and 283 downregulated genes. Red and green colors represent upregulated and downregulated genes, respectively. **(B)** Clustering analysis of DEGs and samples. The color scale bar for heat intensity indicates Log_2_(Fold Change); up and down indicate upregulated and downregulated genes in ROP18-expressing cells, respectively. Columns, samples; rows, DEGs. The samples were grouped into two distinct clusters: ROP18-expressing group and control group. **(C)** qRT-PCR validation of the RNA-seq results. The expressional trends of the examined DEGs were similar between qRT-PCR and RNA-seq results. Blue and green colors represent the result of RNA-seq and qRT-PCR, respectively.

Each sequenced sample had > 119 million raw reads and 110 to 111 million clean reads. Also, 98% and 92% clean reads have met the sequencing quality standards of Q20 and Q30, respectively, demonstrating the high quality of RNA-seq data. Approximately 85%–86% clean reads were mapped to reference human genome (Version: hg38) and 71%–72% clean reads were aligned against reference human genes. A total of 22,460 genes were detected in the HEK293T cells, of which 283 and 467 genes had decreased and increased expression, respectively (**Figure 2A**). Details of the DEGs are shown in **Supplementary Table S2**. Clustering analysis of gene expression clearly separated the data into two clusters (ROP18-expressing cell cluster and control cell cluster), showing the distinct transcriptomic profiles between ROP18 expressing cells and non-ROP18 expressing cells (**Figure 2B**). The RNA-seq data were validated by examining the level of expression of 20 DEGs using qRT-PCR and the results obtained by qRT-PCR and RNA-seq were consistent (**Figure 2C**). Analysis of AS events showed that ROP18 had no significant impact on the SE, A5SS, A3SS, and MXE; however, RI event was significantly increased in ROP18-expressing cells (**Table 1**).

**Table 1:** The number of alternative splicing events in ROP18-expressing compared to non-expressing (control) HEK239T cells.

Sample	SE	MXE	A5SS	A3SS	RI
Control_1	56,027	12,900	5,283	5,419	5,992
Control_2	55,326	12,675	5,215	5,401	5,954
Control_3	50,519	11,138	5,167	5,285	5,985
ROP18_1	52,504	11,444	5,161	5,349	6,043
ROP18_2	55,771	12,523	5,274	5,485	6,100
ROP18_3	50,314	10,772	5,142	5,296	6,036
*P* value of T test	0.665	0.431	0.611	0.911	0.024

SE, skipped exon; A5SS, alternative 5’ splicing site; A3SS, alternative 3’ splicing site; MXE, mutually exclusive exons; RI, retained intron.

### Pathway Enrichment Analysis of DEGs

**Figure 3:**
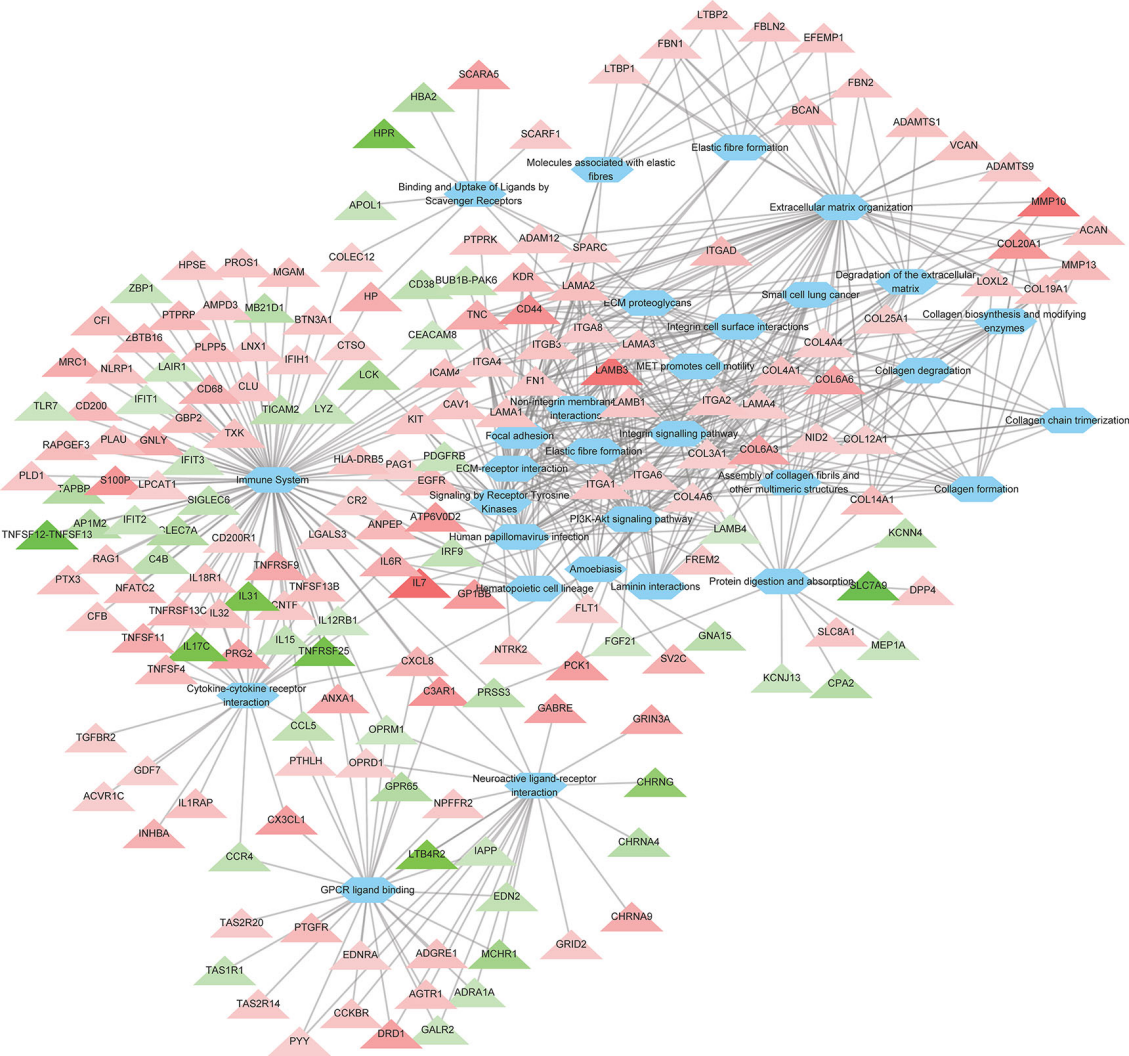
The relationship between the DEGs and the top 30 enriched pathways. A total of 186 DEGs were linked to the top 30 pathways. Triangles represent the DEGs and ovals represent the pathways. Red and green triangles represent upregulated and downregulated DEGs, respectively. The details of DEGs are listed in **Supplementary Table S2**.

To further investigate the the cellular functions that were significantly altered by ROP18 of *T. gondii* RH strain, pathway enrichment analysis was performed. As shown in **Supplementary Table S3**, the DEGs were significantly enriched in 129 pathways. The top 30 enriched pathways were extracellular matrix (ECM) organization, ECM-receptor interaction, ECM proteoglycans, integrin cell surface interactions, degradation of the ECM, focal adhesion, laminin interactions, integrin signalling pathway, non-integrin membrane-ECM interactions, immune system, PI3K-Akt signaling pathway, collagen formation, protein digestion and absorption, assembly of collagen fibrils and other multimeric structures, collagen chain trimerization, cytokine-cytokine receptor interaction, collagen degradation, amoebiasis, hematopoietic cell lineage, binding and uptake of ligands by scavenger receptors, MET activates PTK2 signaling, elastic fibre formation, human papillomavirus infection, small cell lung cancer, molecules associated with elastic fibres, collagen biosynthesis and modifying enzymes, MET promotes cell motility, neuroactive ligand-receptor interaction, GPCR ligand binding, and signaling by receptor tyrosine kinases. All the top 30 pahtways were upregulated by ROP18. The details of the relationship between the DEGs and the top 30 pathways are shown in **Figure 3** and **Supplementary Table S3**.

### Disease Pathway Enrichment Analysis of DEGs

The significantly enriched disease pathways were congenital malformations, congenital malformations of the musculoskeletal system, cardiovascular diseases, immune system diseases, nervous system diseases, eye disease, vascular diseases, epidermolysis bullosa, junctional, atypical hemolytic uremic syndrome, congenital malformations of skin, hematologic diseases, inherited thrombophilia, musculoskeletal diseases, and primary immunodeficiency. Most of these disease related pathways were dominated by upregulated genes. The relationships between DEGs and disease-related pathways are shown in **Supplementary Table S4**.

### GO Enrichment and Transcripton Factor Analysis of DEGs

**Figure 4:**
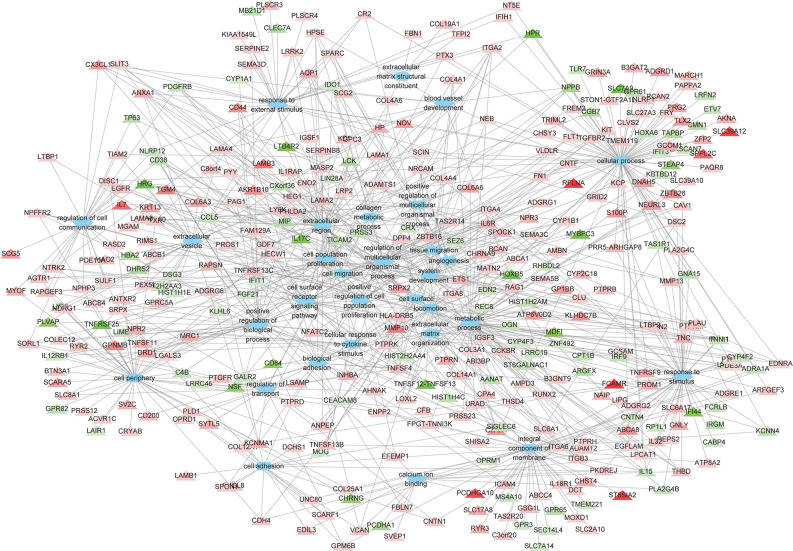
The relationships between the DEGs and the top 30 enriched GO terms. The 373 DEGs were enriched in the top 30 enriched GO terms. Triangles represent the DEGs and ovals represent the GO terms. Red and green triangles represent upregulated and downregulated DEGs, respectively. The details of DEGs are listed in **Supplementary Table S2**.

**Figure 5:**
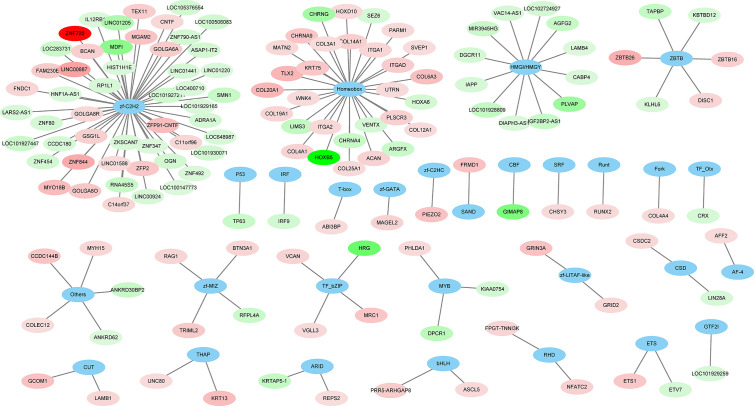
The families of differentially expressed transcription factors (DETFs). A total of 144 DETFs were categorized into 29 transcription families. Blue ovals represent the TF family. Red and green ovals represent TF with increased and decreased expression, respectively. The details of DETFs are listed in **Supplementary Table S2**.

**Figure 6:**
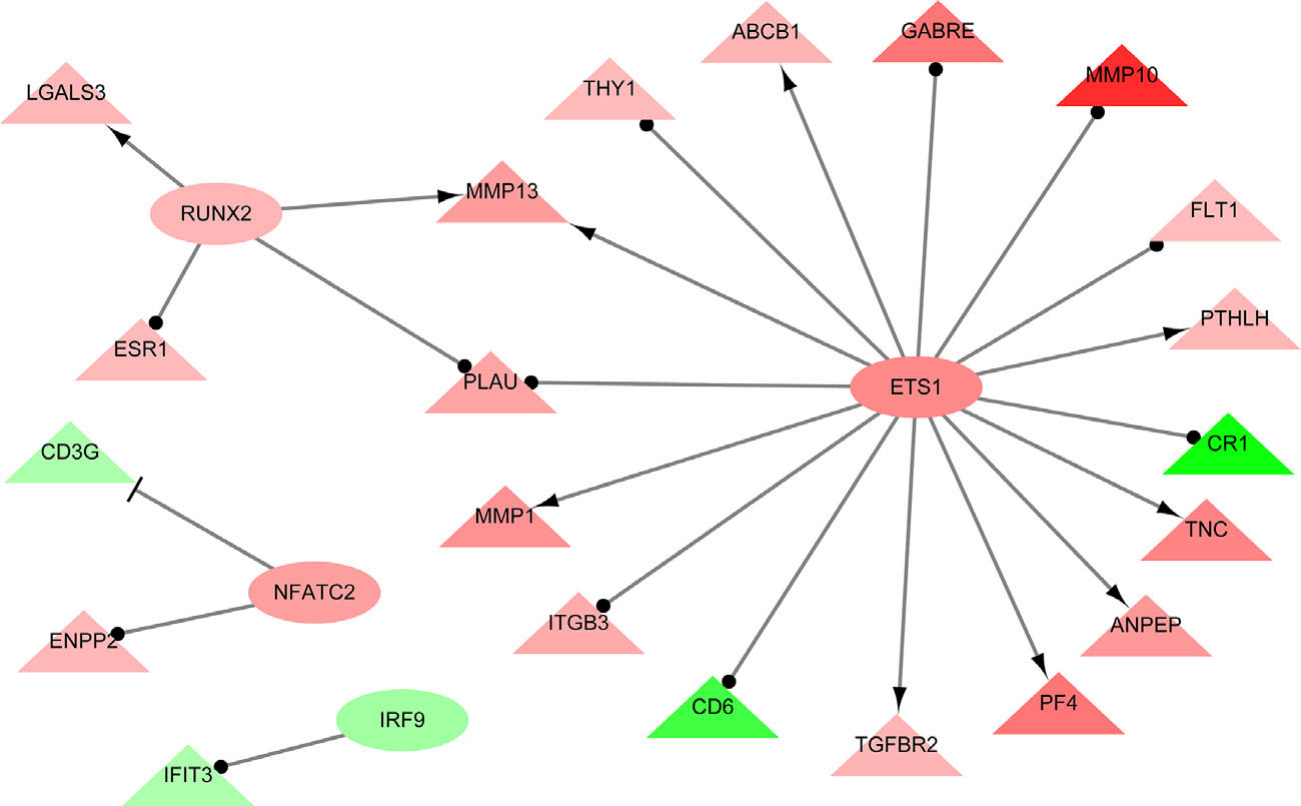
The interaction network showing the relationships between differentially expressed transcription factors (DETFs) and their corresponding target genes. Ovals and triangles represent DETFs and their target genes, respectively. Red and green denote genes with increased and decreased expression, respectively. Arrows with a T-shaped end represent inhibition or repression of gene expression, arrows with a delta-shaped end represent gene activation, and arrows with a dot-shaped end represent unknown regulatory type. Network was constructed using Cytoscape and TRRUST database. The details of DEGs are listed in **Supplementary Table S2**.

A total of 264 GO terms were significantly enriched by DEGs. The top 30 enriched GO terms included twenty-three biological process GO terms (response to external stimulus, regulation of multicellular organismal process, system development, positive regulation of multicellular organismal process, collagen metabolic process, cell adhesion, locomotion, cell surface receptor signaling pathway, cellular response to cytokine stimulus, cellular process, angiogenesis, positive regulation of cell population proliferation, ECM organization, blood vessel development, biological adhesion, regulation of transport, positive regulation of biological process, response to stimulus, cell migration, tissue migration, cell population proliferation, regulation of cell communication, and metabolic process), five cellular component GO terms (integral component of membrane, cell periphery, extracellular region, extracellular vesicle, and cell surface), and two molecular function GO terms (ECM structural constituent and calcium ion binding) (**Figure 4** and **Supplementary Table S5**). We also identifed 144 differentially expressed TFs (DETFs), including 75 upregulated TFs and 69 down-regulated TFs. As shown in **Figure 5**, the DETFs were classed into 29 families. zf-C2H2, Homeobox and HMGI/HMGY were the top 3 families that contained most DETFs altered by ROP18 of *T. gondii*. We identified the target DEGs of DETFs in the TRRUST database, where 16, 4, 2, and 1 DEGs are targeted by ETS1, RUNX2, NFATC2, and IRF9, respectively (**Figure 6**).

## Discussion

In this study, we expressed ROP18 of RH strain in HEK293T cells and studied the resultant effects on the cell transcriptome by using RNA-seq approach. Sequencing of PCMV-N-HA-ROP18 plasmid showed that ROP18 eukaryotic expression plasmid has been successfully constructed, and Western blotting showed that ROP18 was correctly expressed in HEK293T cell (**Figure S1**). As shown in **Figure 1**, no HA-tagged protein was detected in the control cells, however a strong fluorescent signal was detected in HEK293T cells transfected with PCMV-N-HA-ROP18. RNA-seq showed that ROP18 of RH strain decreased the expression of 283 gene but increased the expressions of 467 genes of HEK293T cells (**Figure 2A** and **Supplementary Table S2**). ROP18-expressing cell cluster and control cell cluster were clearly separated into two clusters, indicating the distinct transcriptomic profiles between ROP18 expressing cells and non-ROP18 expressing cells (**Figure 2B**). The qRT-PCR validation showed an agreement between the results obtained by qRT-PCR and RNA-seq (**Figure 2C**), demonstrating the reliability of the RNA-seq data.

The GO enrichment and pathway analyses showed that DEGs were significantly enriched in 129 pathways (**Supplementary Table S3**), and 115 DEGs were linked to 14 KEGG disease pathways (**Supplementary Table S4**). Most of the top 30 enriched pathways were involved in ECM, cell binding and immune response (**Figure 3**). Consistent with the KEGG analysis, most of the top 30 enriched GO terms were also related to ECM, cell binding and immune response (**Figure 4** and **Supplementary Table S5**). These data clearly showed that a large number of ECM-related pathways and GO terms were significantly enriched (**Figures 3** and **4**). These findings are expected because HEK293 cells are frequently used as a model for ECM-interaction studies because they express several β1 integrin containing subunits on their cell surface, which allow them to adhere to a range of ECM proteins (Bodary and McLean, 1990). ECM components are critical scaffolds for adhesive cells, and regulate proliferation, differentiation, and fate of the cells. All these crucial processes contribute to cell migration, cellular communication, inflammation, and histopathology. Alterations in ECM composition, structure, abundance, or expression of ECM genes have been shown to cause or underpin sevreal diseases (Lamande and Bateman, 2020). Given these highly versatile functions of ECM, it is not surprising to see significant alterations in multiple disease-related processes enriched by DEGs. Also, ECM plays a key role in the morphogenesis and regulation of the neural progenitor behavior (Long and Huttner, 2019). We also found that ECM organization and congenital malformation processes were significantly enriched by 47 DEGs (**Figure 3**) and 47 DEGs (**Supplementary Table S4**), respectively. Most of the DEGs were upregulated by ROP18. Whether alterations in the expression of genes related to ECM or tissue defects caused by ROP18 contribute to the prenatal congenital pathologies that occur in the fetus who become infected during pregnancy remains to be investigated.

ECM modulates the activities of growth factors and cytokines (Schonherr and Hausser, 2000). Also, upregulation of ECM components has been linked to inflammatory responses (Sorokin, 2010; Herrera et al., 2018). We identified 88 immune-related DEGs in ROP18-expressing cells, including 61 upregulated and 27 downregulated genes (**Figure 3**). The enriched innate immune system pathway was alterd by 28 upregulated genes (*CR2*, *LPCAT1*, *IFIH1*, *LGALS3*, *AMPD3*, *HPSE*, *CLU*, *PLD1*, *PROS1*, *CFB*, *NLRP1*, *TXK*, *MGAM*, *PLAU*, *PTX3*, *PLPP5*, *NFATC2*, *CFI*, *PTPRB*, *ANPEP*, *CD68*, *HP*, *GNLY*, *PRG2*, *ATP6V0D2*, *S100P*, *C3AR1*, and *CD44*) and 11 downregulated genes (*LCK*, *CLEC7A*, *MB21D1*, *PRSS3*, *TICAM2*, *C4B*, *ZBP1*, *LYZ*, *TLR7*, *LAIR1*, and *CEACAM8*) (**Supplementary Table S3**). Also, 24 genes of cytokine-cytokine receptor interaction pathway were significantly altered (**Figure 3** and **Supplementary Table S3**), including 17 upregulated genes (*GDF7*, *ACVR1C*, *TGFBR2*, *CNTF*, *IL18R1*, *IL1RAP*, *TNFSF13B*, *IL32*, *TNFSF4*, *TNFRSF13C*, *CXCL8*, *INHBA*, *IL6R*, *TNFSF11*, *TNFRSF9*, *CX3CL1*, and *IL7*) and 7 downregulated genes (*TNFRSF25*, *IL17C*, *IL31*, *CCR4*, *CCL5*, *IL15*, and *IL12RB1*). In these differentially expressed cytokine-related genes, four of them regulate the chemotaxis of immune cells, including *CXCL8*, *CXCL1*, *CCR4*, and *CCL5*. These chemotaxis-related genes have several biological and immunological functions. Maintaining a balanced immune response during *T. gondii* infection is essential in order to limit the parasite proliferation, while at the same time protects the host from the adverse effects of excessive inflammatory pathologies (Chousterman et al., 2017). The CCL5 which regulates the migration of eosinophils and regulatory T cells (Griffith et al., 2014) was downregulated by ROP18. However, CXCL8 and CXCL1 that regulate the chemotaxis of CD8^+^ effector T cells, resident monocytes, microglia, CD8^+^ effector-memory T cells, and T cells were significantly upregulated by ROP18. Thus, it is possible that ROP18 contributes to the recruitment of host immune cells to the infection site.

We also found that DEGs were enriched in several disease pathways. Chorioretinitis is a common manifestation in ocular toxoplasmosis, and a correlation exists between ROP18 allele type and the severity of ocular inflammatory response (Sanchez et al., 2014). As shown in **Supplementary Table S4**, ROP18 altered the expressions of 15 eye disease-related genes, including *EFEMP1*, *SLC7A14*, *MIP* (major intrinsic protein of lens fibe), *COL25A1*, *CFB*, *SLC38A8*, *CFI*, *RIMS1*, *CABP4*, *RP1L1*, *CRYAB*, *PROM1*, *CRX*, *KCNJ13*, and *VCAN*. Previous studies showed that *EFEMP1* (Lin et al., 2018; Thompson et al., 2019), *SLC7A14* (Jin et al., 2014), and *RP1L1* (Albarry et al., 2019) are linked to macular degeneration or retinitis pigmentosa; *COL25A1*, which encodes a membrane associated collagen, is associated with oculomotor neuron development (Shinwari et al., 2015). Also, *RP1L1* (Fujinami-Yokokawa et al., 2019), *PROM1* (Fujinami et al., 2020), *CRX* (Fujinami-Yokokawa et al., 2020), *CFI* and *CFB* (Rathi et al., 2017; Shahulhameed et al., 2020), and *KCNJ13* (Toms et al., 2019) have been linked to retinopathy. Additionally, *SLC38A8* contributes to congenital nystagmus (Weiner et al., 2020), and *RIMS1* and *CABP4* are associated with dystrophy (Sisodiya et al., 2007) and synaptic disorder of cone-rod (Littink et al., 2009), respectively. Furthermore, alteration of *CRYAB* is associated with cataract (Molnar et al., 2019), and *VCAN* is associated with vitreoretinal degeneration (Tang et al., 2019). Most of these eye disease-related genes were upregulated in HEK293T cells by ROP18 (**Supplementary Table S3**). Whether the same genes are also altered in other cell lines such as occular cell types remains to be determined. A previous study showed that the expression of IFN-γ and IL-1β was not significantly influenced by ROP18 in peripheral blood mononuclear cells collected from patients with ocular toxoplasmosis (Hernandez-de-Los-Rios et al., 2019). Our results also showed that the expression of IFN-γ and IL-1β was not significantly influenced by the expression of ROP18 protein in HEK293T cells.

Recent studies show that *T. gondii* infection can induce significant structural, functional and metabolic changes in the brain microvascular endotehlial cells (Al-Sandaqchi et al., 2018; Hu et al., 2018; Ma et al., 2019; Al-Sandaqchi et al., 2020; Harun et al., 2020a; Harun et al., 2020b) and can change the neuron subpopulations (Odorizzi et al., 2010). However, the exact mechanisms of behavioral abnormalities and change in the subpopulations of neurons induced by *T. gondii* infection remains to be clearly defined. A previous study revealed a role for ROP18 in increased neural apoptosis and encephalitis during *T. gondii* infection (An et al., 2018). Although HEK293T cells are not drieved from brain, our transcriptomic analysis showed that ROP18 can alter the expressions of genes involved in several neural activity-related pathways, neuron differentiation and development processes. We found that the neural activity-related pathways/GO terms were significantly enriched in HEK293T cells following expression of ROP18 protein. Neuroactive ligand-receptor interaction was enriched by 23 DEGs, including 13 upregulated genes and 10 downregulated genes (**Figure 5** and **Table Supplementary S3**). Nervous system diseases were also enriched by 23 upregulated genes and 13 downregulated genes (**Supplementary Table S4**). GO enrichment analysis showed that neuronal cell body and neuron differentiation process were significantly altered by 10 DEGs and 13 DEGs, respectively; cell morphogenesis involved in neuron differentiation was significantly altered by 6 upregulated genes and 3 downregulated genes; and regulation of neuron differentiation was significantly altered by 5 upergulated genes and 1 downregulated genes (**Supplementary Table S5**). Although the impact of ROP18 on neurons remains to be determined, our results offer preliminary results for further investigation of the effect of ROP18 on the neurobiology of cerebral toxoplasmosis.

RNA-seq analysis has been used to detect AS events (Filichkin et al., 2010; Feng et al., 2013; Shen et al., 2014). Our previous study showed that ROP17 of *T. gondii* can modify host AS events (Li et al., 2019) which have significant roles in various biological processes (Blencowe, 2006; Baralle and Giudice, 2017). We investigated the role of ROP18 in the regulation of host AS events by comparing five AS events, including SE, A5SS, A3SS, MXE, and RI, in ROP18-expressing and control cells. As shown in **Table 1**, RI event was significantly increased in ROP18-expressing cells. RI is a type of AS envent that can introduce functional elements to the protein (Buckley et al., 2011) or results in the degradation of mRNA by RNA surveillance mechanism (Belgrader et al., 1994). This result suggests that ROP18 can influence host biological processes *via* altering the RI event within the host cells. The exact mechanism by which ROP18 alters RI event is unknown, however, we found that U2 small nuclear RNA auxiliary factor 1 like 5 (U2AF1L5) was significanlty downregulated (Log_2_FC = –1.135, FDR corrected *P* value = 0) in ROP18-expressioning cells. The U2AF1L5 seems to participate in mRNA splicing according to annotation in NCBI database.

Analysis of the regulatory networks between DEGs and TFs is important for elucidating the role of ROP18 in regulating the host biological processes. ROP18 protein upregulated the expressions of 75 TFs, but downregulated the expressions of other 69 TFs in HEK293T cells, showing the significant impact of ROP18 on the expression of TFs. The TFs altered by ROP18 were classed into 29 families, and the zf-C2H2, Homeobox and HMGI/HMGY families were the top families with the most DETFs (**Figure 6**). These results suggest a marked influence of ROP18 on the expression of TFs belonging to these three TF families. Most DETFs of Homeobox family were upregulated, however all DETFs of HMGI/HMGY family were downregulated (**Figure 5**), suggesting that ROP18 could have a suppressive effect on members of the HMGI/HMGY family. By searching TRRUST database, we identified four DETFs, including ETS1, RUNX2, NFATC2, and IRF9, which target 16, 4, 2, and 1 DEGs, respectively (**Figure 6**). ETS1 induces the expression of *MMP13* (Ghosh et al., 2012), *ABCB1* (Kars et al., 2010), *PTHLH* (Dittmer et al., 1994), *TNC* (Jinnin et al., 2004), *ANPEP* (Petrovic et al., 2003), *PF4* (Okada et al., 2003), *TGFBR2* (Kopp et al., 2004), and *MMP1* (Mix et al., 2007). RUNX2 enhances the expression of *LGALS3* (Zhang et al., 2009) and *MMP13* (Wang et al., 2004). NFATC2 suppresses the expression of *CD3G*. The expression of these target genes is consistent with the regulatory functions of the DETFs, suggesting that ROP18 modifies host gene expression *via* altering the expression of TFs. Analysis of the interaction between ROP18 and host TFs may elucidate the interplay between ROP18 and cellular processes. Previous studies showed that ROP18 interacts with several TFs, including SOX6, SPDEF, HMGN1, ATF3, MLLT10, DNMT3L, MYCN, MXD4, TAF12, EPAS1, CNBP, HMGA1, ATM, TBX3, ZNF148, p65, p53, ATF6B, and SMAD1 (Cheng et al., 2012; Du et al., 2014; Yang et al., 2017; Xia et al., 2018). Interestingly, the expressions of these interacting TFs were not significantly altered by ROP18. However, by searching TRRUST database, we found that MYCN activates the expressions of CD44 and NDRG1, EPAS1 activates the expression of FLT1, and HMGA1 activates the expression of CD44. In this study, CD44 (Log2FC = 2.5, FDR corrected *P*-value = 1.23E-30), NDRG1 (Log2FC = 1.1, FDR corrected *P*-value = 0) and FLT1 (Log2FC = 1, FDR corrected *P*-value = 1.16E-36) were upregulated by ROP18 stimulation. The expression of these target genes is consistent with the regulatory functions of the MYCN, EPAS1, and HMGA1. Whether these regulatory effects depend on the phosphokinase activity of ROP18 remains to be elucidated.

In our study, the cell cycle process was not significantly affected by ROP18 at the gene transcriptional level. However, another effector protein, ROP16, plays a significant role in host cell cycle (Chang et al., 2015). The difference between these two virulence-associated proteins (ROP16 and ROP18) in the effect on host cell cycle may be attributed to differences in their host target genes. In a previous study, ROP18 of RH strain was found to interact with 492 host proteins (Xia et al., 2018). In our study, only a few of these interacting proteins (including upregulated DDX60, COL6A3, PTPRK, and RCAN2; downregulated LYPD5, KIR3DX1, NPPB, and TNNI1) were significantly altered at the gene expression level. This difference might be attributed to variations in the behavior of the transfected host cells. Both ROP17 and ROP18 are secretory proteins of the ROP2 family (El Hajj et al., 2006) and have a similar location within the host cell (Etheridge et al., 2014). By comparing the host transcriptional responses to ROP17 (Li et al., 2019) and ROP18 in the present study, we identified 110 and 276 genes whose expression was decreased or increased, respectively, in both ROP17 and ROP18. This similarity in the location inside the host cell and in the effect on host cell transcriptome is consistent with the fact that ROP17 and ROP18 share some host cell targets (Etheridge et al., 2014). ROP5 forms complexes with ROP18 and ROP17 to mediate the parasite survival in mice (Etheridge et al., 2014). A link between ROP18 allele type and virulence in mice has been reported (Sanchez et al., 2014) and the combination of ROP18/ROP5 allele types was found to be even more predictive of *T. gondii* virulence in mice (Shwab et al., 2016). Given the interaction and overlap between the functions of ROP proteins, studying the effect of simultaneous expression of ROP5, ROP16, ROP17, and ROP18 on the host cell transcriptional reprogramming may improve the understanding of the virulence mechanism of *T. gondii*.

## Conclusion

This study presents the first RNA-Seq-based analysis of the transcriptomic responses of HEK239T cells to ROP18 expression. We identified 22,460 host genes, and the expression of 750 genes was significantly altered by ROP18, including 467 upregulated genes and 283 downregulated genes. The functions of significantly altered genes were mainly involved in ECM organization, immune responses and disease processes. ROP18 also alters the expression of 144 TFs belonging to 29 TF families and increased the RI pattern of AS. Our data revealed several potential new roles of ROP18 in the transcriptional regulation of host cells. Further investigations of the effects of a catalytic inactive mutant of ROP18 on the host cell transcriptome and using different cell lines (e.g. neurons and immune cells) will deepen our understanding of *T. gondii* interactions with the host cell processes. Also, using methods such as siRNA and gene editing to alter ROP18 protein expression can improve the evaluation of the effects of ROP18 protein with the concomitant entry of live parasites.

## Data Availability Statement

The datasets presented in this study can be found in online repositories. The names of the repository/repositories and accession number(s) can be found at https://www.ncbi.nlm.nih.gov/ (SRR7825256), https://www.ncbi.nlm.nih.gov/ (SRR7825257), https://www.ncbi.nlm.nih.gov/ (SRR7825258), https://www.ncbi.nlm.nih.gov/ (SRR12130694), https://www.ncbi.nlm.nih.gov/ (SRR12130695), and https://www.ncbi.nlm.nih.gov/ (SRR12130696).

## Author Contributions

J-JH, HME, and X-QZ conceived and designed the study and critically revised the manuscript. J-XL and J-JH performed the experiment, analyzed the transcriptomic data, and drafted the manuscript. JM and X-PX helped in data analysis and manuscript revision. All authors contributed to the article and approved the submitted version.

## Funding

Project support was provided by the National Key Research and Development Program of China (Grant No. 2017YFD0500403), the National Natural Science Foundation of China (Grant No. 31902291), and the International Science and Technology Cooperation Project of Gansu Provincial Key Research and Development Program (Grant No. 17JR7WA031).

## Conflict of Interest

The authors declare that the research was conducted in the absence of any commercial or financial relationships that could be construed as a potential conflict of interest.

## Supplementary Material

Supplementary Table 1 The primers used in the qRT-PCR analysis.

Supplementary Table 2 Differentially expressed genes altered by ROP18 of *T. gondii* RH strain.

Supplementary Table 3 Pathway enrichment analysis of the differentially expressed genes altered by ROP18 of *T. gondii* RH strain.

Supplementary Table 4 Analysis of KEGG disease related pathway involving the differentially expressed genes altered by ROP18 of *T. gondii* RH strain.

Supplementary Table 5 GO enrichment analysis of the differentially expressed genes altered by ROP18 of *T. gondii* RH strain.

Supplementary Figure 1 Western blotting analysis using anti-HA tag antibody detects the ROP18 protein in HEK293 cells transfected with PCMV-N-HA-ROP18. PL, PageRuler™ Prestained Protein Ladder; C, Extract of control HEK293 transfected with the plasmid PCMV-N-HA; 18, Extract of HEK293 transfected with the plasmid PCMV-N-HA-ROP18.
