# Supplementary figures and images for: ROP18-Mediated Transcriptional Reprogramming of HEK293T Cell Reveals New Roles of ROP18 in the Interplay Between Toxoplasma gondii and the Host Cell
Source: Front Cell Infect Microbiol. 2020 Nov 30;10:586946. doi: 10.3389/fcimb.2020.586946 (PMC7734210; doi:10.3389/fcimb.2020.586946)

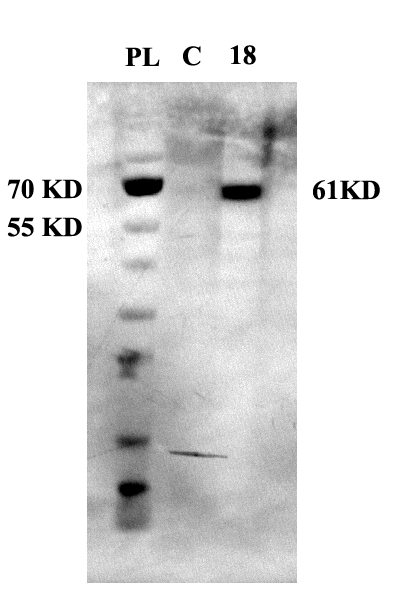

Supplement: Supplementary Figure 1 — Western blotting analysis using anti-HA tag antibody detects the ROP18 protein in HEK293 cells transfected with PCMV-N-HA-ROP18. PL, PageRuler™ Prestained Protein Ladder; C, Extract of control HEK293 transfected with the plasmid PCMV-N-HA; 18, Extract of HEK293 transfected with the plasmid PCMV-N-HA-ROP18. [file Image_1.tif]
